# Quality of surgical antibiotic prophylaxis using E-prescrption conditioning

**DOI:** 10.1186/1753-6561-5-S6-O44

**Published:** 2011-06-29

**Authors:** JFC Rodrigues, A Duarte, C Palos, A Casado, C Santos, F Fernandez-Llimos

**Affiliations:** 1 Pharmacyst, Lisbon, Portugal; 2 ICU Physician, Hospital Da Luz, Lisbon, Portugal; 3 Head of Pharmaceutical Department, Hospital Da Luz, Lisbon, Portugal; 4 iMed.UL, Faculty of Pharmacy, University of Lisbon, Lisbon, Portugal

## Introduction / objectives

Hospital da Luz is a paper-free hospital. Antibiotic prescription is made electronically and is automatically conditioned by both context and duration. We aimed to assess the pattern of use of antibiotics in surgical prophylaxis in a general hospital.

## Methods

Prophylactic antibiotic prescriptions for patients undergoing surgery in January 2011 were extracted from the medical records. Variables collected included: surgical procedure, prophylactic antibiotic prescribed (ATC code), surgery classification (clean, clean contaminated, contaminated and dirty), and prophylaxis duration. A descriptive statistical analysis and cross-tabulations (chi-square) were performed.

## Results

611 prophylactic antibiotics were initiated for patients with an average age of 49.6 years (SD=16.6), (60.6% females). Surgeries were classified as: clean (39.1%), clean contaminated (29.4%), contaminated (2.1%) and dirty (1.2%). Most prescribed antibiotics were: first-generation cephalosporins (83.6%), second-generation cephalosporins (8.0%), imidazole derivatives (4.6%) and quinolones (1.8%). Antibiotic administration was exclusively intraoperative in 50.8% of the cases. 34.5% of the prescriptions were extended for 24 hours, 12.4% for 48 hours and 2.3% for more than 48 hours. Statistical association between surgery classification and treatment duration was found (chi-square p=0.010). Quinolones were prescribed only in urological surgery and imidazoles were prescribed only in colorectal surgery.

## Conclusion

Antibiotic prophylaxis using e-prescription conditioning resulted on adequate compliance with guidelines, although opportunities for improvement were found.

## Disclosure of interest

None declared.

